# Imaging cytoplasmic cAMP in mouse brainstem neurons

**DOI:** 10.1186/1471-2202-10-29

**Published:** 2009-03-27

**Authors:** SL Mironov, E Skorova, G Taschenberger, N Hartelt, VO Nikolaev, MJ Lohse, S Kügler

**Affiliations:** 1 DFG-Center of Molecular Physiology of the Brain, Department of Neuro- and Sensory Physiology, Humboldtallee 23, Georg-August-University, 37073 Göttingen, Germany; 2 University Medical Center Göttingen, Department of Neurology, Waldweg 33, 37073 Göttingen, Germany; 3 Institute of Pharmacology and Toxicology, University of Würzburg, Versbacher Str. 9, 97078 Würzburg, Germany

## Abstract

**Background:**

cAMP is an ubiquitous second messenger mediating various neuronal functions, often as a consequence of increased intracellular Ca^2+ ^levels. While imaging of calcium is commonly used in neuroscience applications, probing for cAMP levels has not yet been performed in living vertebrate neuronal tissue before.

**Results:**

Using a strictly neuron-restricted promoter we virally transduced neurons in the organotypic brainstem slices which contained pre-Bötzinger complex, constituting the rhythm-generating part of the respiratory network. Fluorescent cAMP sensor Epac1-camps was expressed both in neuronal cell bodies and neurites, allowing us to measure intracellular distribution of cAMP, its absolute levels and time-dependent changes in response to physiological stimuli. We recorded [cAMP]_i _changes in the micromolar range after modulation of adenylate cyclase, inhibition of phosphodiesterase and activation of G-protein-coupled metabotropic receptors. [cAMP]_i _levels increased after membrane depolarisation and release of Ca^2+ ^from internal stores. The effects developed slowly and reached their maximum after transient [Ca^2+^]_i _elevations subsided. Ca^2+^-dependent [cAMP]_i _transients were suppressed after blockade of adenylate cyclase with 0.1 mM adenylate cyclase inhibitor 2'5'-dideoxyadenosine and potentiated after inhibiting phosphodiesterase with isobutylmethylxanthine and rolipram. During paired stimulations, the second depolarisation and Ca^2+ ^release evoked bigger cAMP responses. These effects were abolished after inhibition of protein kinase A with H-89 pointing to the important role of phosphorylation of calcium channels in the potentiation of [cAMP]_i _transients.

**Conclusion:**

We constructed and characterized a neuron-specific cAMP probe based on Epac1-camps. Using viral gene transfer we showed its efficient expression in organotypic brainstem preparations. Strong fluorescence, resistance to photobleaching and possibility of direct estimation of [cAMP] levels using dual wavelength measurements make the probe useful in studies of neurons and the mechanisms of their plasticity. Epac1-camps was applied to examine the crosstalk between Ca^2+ ^and cAMP signalling and revealed a synergism of actions of these two second messengers.

## Background

cAMP is an ubiquitous second messenger which regulates a wide variety of cellular events and processes ranging from metabolism and gene expression, cell division and migration, exocytosis and secretion, to memory formation and cardiac contractility [1]. On the one hand, cAMP-signalling cascades are associated with many vital functions in the CNS that can be exemplified by cAMP-related changes in neuronal activity [2], dendritic and axonal growth [3], spine function [4], synaptic activity and neuronal plasticity [5], and gene expression [6] etc. On the other hand, inadequate cAMP signalling may lead to long-term disturbances of neuronal functions [7]. cAMP-dependent enzymes are well characterized in terms of their affinity, kinetics and mechanisms of their modulation [1]. In order to understand how cAMP-dependent signalling mechanisms operate in living neurons, appropriate tools are needed to monitor spatial and time-dependent changes of cAMP levels.

Direct imaging of cAMP became possible only recently. Two classes of probes have been developed – one based on protein kinase A (PKA) and utilizing fluorescence resonance energy transfer (FRET) between fluorescein and rhodamine [8] or CFP and YFP [9], and the other exploiting the exchange protein directly activated by cAMP, namely Epac [10-12]. PKA-based cAMP probes have slow response times, making them not very suitable for monitoring the rapid changes in [cAMP]_i _which occur in living cells. Originally designed Epac has good sensitivity and temporal resolution [10,11]. Both features are conserved in Epac-1-camps [12], its smaller size allowing it to be incorporated into viral vectors with limited genome sizes such as AAV. In comparison with the common methods of transfection in primary cultures [13] and organotypic slices [14], the efficacy of viral gene transfer in postmitotic neurons is much higher. We recently used adeno-associated viruses (AAV) driving transgene expression by the strictly neuron-specific synapsin 1 gene promoter [15] to selectively express GFP in neurons in organotypic brainstem slices [16] containing pre-Bötzinger complex (preBötC), the latter constituting the rhythm-generating part of the respiratory network [17-19]. An important step in further understanding spatial and functional organisation of the respiratory network now is the expression of fluorescent sensors in respiratory neurons and measuring their responses to physiological stimuli.

Respiratory activity is crucially dependent on cAMP signalling, which can be dynamically modulated in specific physiological and pathological conditions [17]. In this study we aimed to specifically target Epac1-camps to brainstem neurons. We characterised the sensitivity and specificity of the sensor to report intraneuronal cAMP. We measured [cAMP] changes after modulation of adenylate cyclase (AC), phosphodiesterase (PDE) and activation of G-protein-coupled metabotropic receptors. [cAMP] changes were tightly related to calcium transients, which are important for respiratory rhythmogenesis [20]. Both depolarisation-triggered Ca^2+ ^entry and agonist-induced Ca^2+ ^release from endoplasmic reticulum (ER) led to cAMP increase caused by enhancement of AC activity by calcium. Paired calcium increases produced a non-linear summation of subsequent [cAMP]_i _increases. This was abolished after PKA inhibition, thus indicating an important role for phosphorylation in the dynamic interplay between Ca^2+ ^and cAMP, previously documented in non-excitable cells [21].

## Results

**Figure 1:**
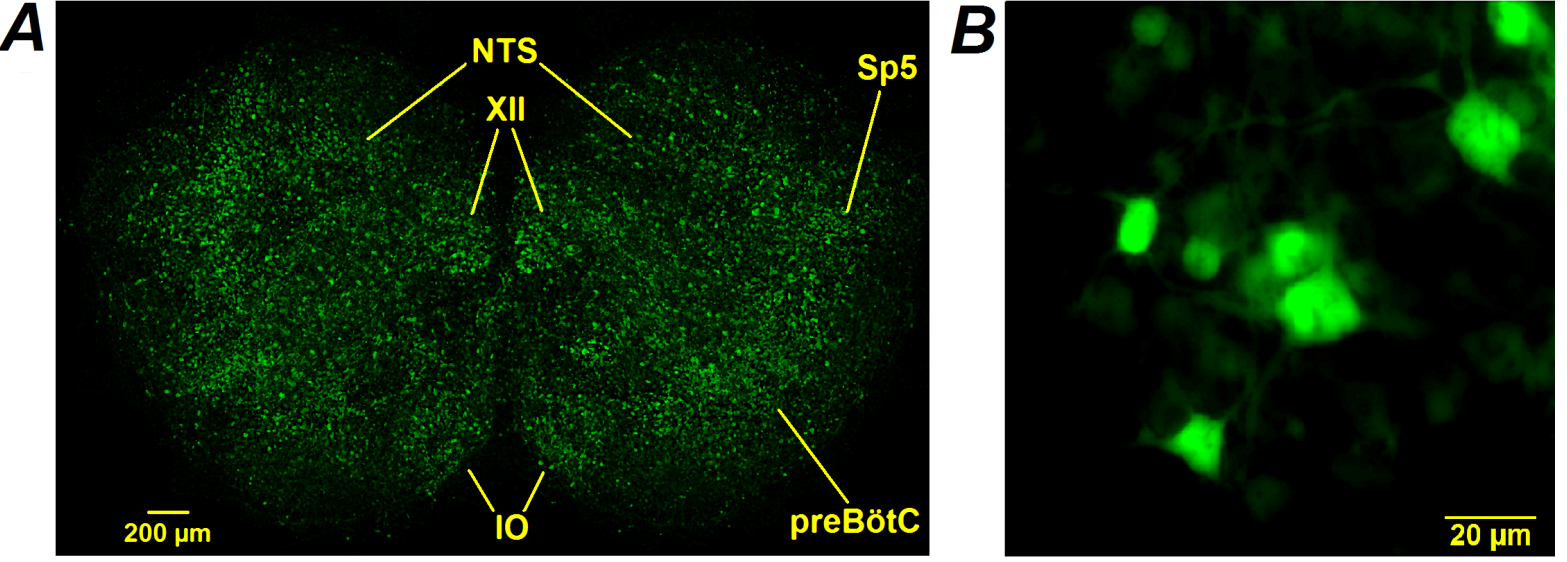
**Expression of neuron-specific Epac1-camps**. Representative original images of AAV-Epac1-camps-transduced organotypic slice (A) which contained pre-Bötzinger complex (preBötC) and other characteristic nuclei (XII – Nucleus hypoglossus, NTS – Nucleus tractus solitarii, IO – inferior olive, Sp5 – spinal nucleus) and neurons in preBötC (B). The images were acquired at ×10 (A) and ×40 (B) magnification.

Application of the AAV-Epac1-camps vector resulted in expression of the sensor in many neurons per living brainstem slice (Fig. 1A). These neurons were arranged into groups resembling the nuclei characteristic for this section of the preBötC-containing brainstem [19]. Neuronal somata displayed brighter fluorescence but neuronal processes were also distinctly resolvable (Fig. 1B). These acquisitions sharply contrast to images obtained using conventional fluorescent calcium probes such as fluo-3 where only the somata of neurons were clearly visible [16].

**Figure 2:**
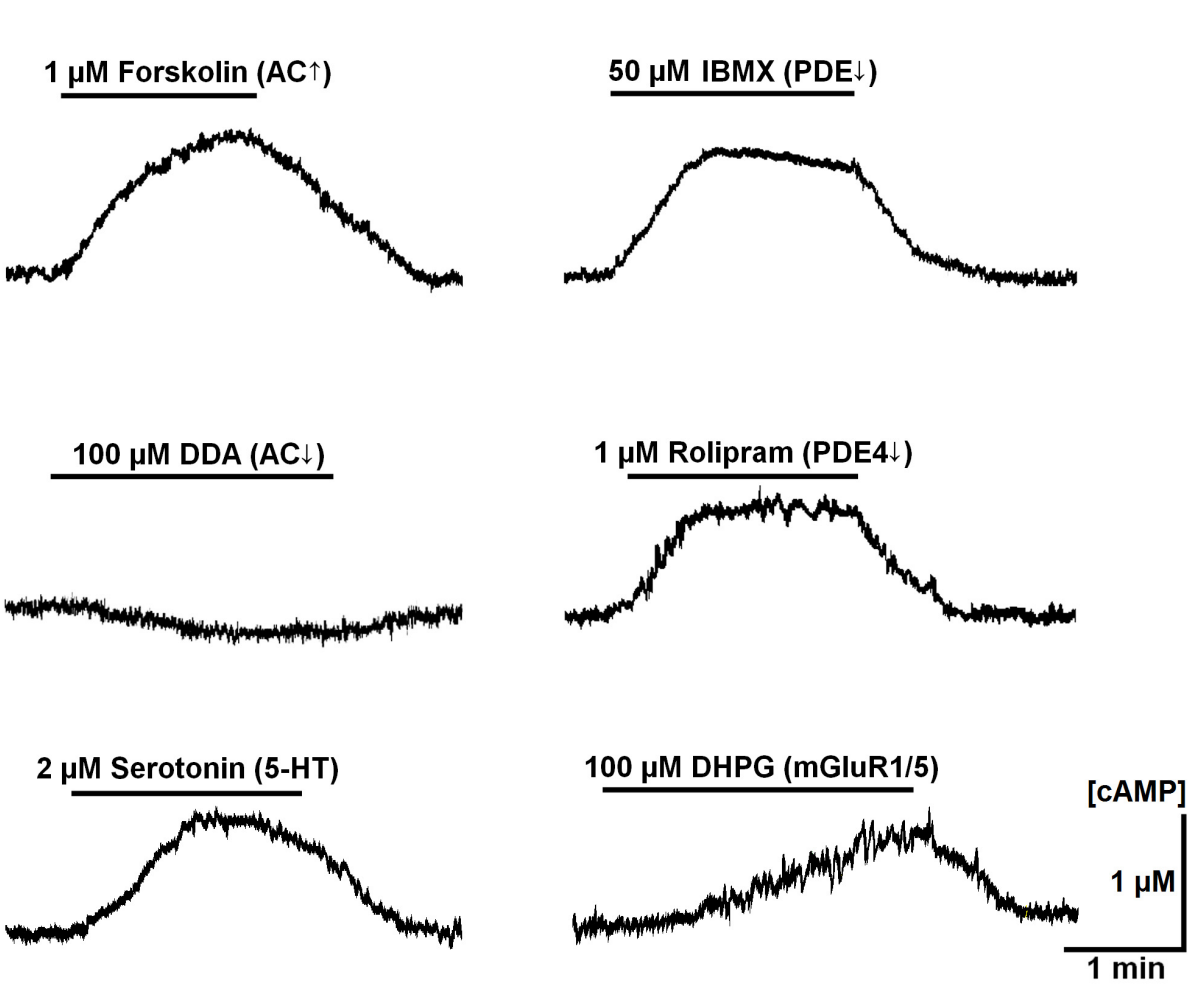
**Characteristic [cAMP] changes in preBötC neurons**. Shown are representative responses (for the statistics, see Table 1) due to modulation of activity of adenylate cyclase, phosphodiesterase and activation of metabotropic receptors. AC was stimulated with forskolin and inhibited with 2'5'-dideoxyadenosine (DDA), PDE was antagonized with IBMX and rolipram, and Group I metabotropic glutamate receptors were activated with (S)-3,5-dihydroxyphenylglycine (DHPG). Fluorescence signals were averaged over the soma of neurons and transformed into cAMP concentrations as described in Methods.

The ability of Epac1-camps to register [cAMP]_i _changes was first examined in tests where activities of adenylate cyclase (AC) and phosphodiesterase (PDE) were modulated. Applications of 1 μM forskolin (a specific AC activator), 50 μM isobutylmethylxanthine (IBMX, non-specific PDE inhibitor), 1 μM rolipram (a specific inhibitor of PDE4 [22]), and 0.1 mM 2'5'-dideoxyadenosine (DDA, membrane-permeable AC inhibitor) induced [cAMP]_i _changes in line with the presumed drug targets (Fig. 2, Table 1). Effects induced by the drugs were reversible and the responses could be repeatedly elicited after washing-out for 10 to 15 min. Rolipram was the most effective among PDE inhibitors – the increases in [cAMP]_i _induced by PDE3 inhibitor milrinone (1 μM) and PDE2 inhibitor EHNA (10 μM) were 0.58 ± 0.05 and 0.21 ± 0.06 μM, respectively. These effects were smaller than those induced by rolipram (Table 1) and their magnitude correlates well with the efficacy of the drugs in cardiomyocytes [23]. The greater potency of rolipram thus can explain its stimulating effect on the respiratory motor output *in vivo *[19]. As exemplified by the two bottom panels in Fig. 2, activation of G-protein-coupled receptors produced delayed cAMP increases, showing the actions of serotonin (5-HT) and the mGluR1/5 agonist (S)-3,5-dihydroxyphenylglycine (DHPG). These responses are consistent with the activation of metabotropic serotonin and glutamate receptors, respectively.

**Table 1:** Mean [cAMP]_i _changes observed after modulation of cAMP signalling pathway and activation of G-protein coupled glutamate and serotonin receptors

Forskolin 1 μM	IBMX 50 μM	DDA 100 μM	Rolipram 1 μM	DHPG 100 μM	5-HT 2 μM
1.62 ± 0.12	1.45 ± 0.07	-0.12 ± 0.05	1.19 ± 0.08	0.74 ± 0.07	0.81 ± 0.05

Mean data ± S. E. M. were obtained in 4 to 8 neurons in four different preparations and are presented as changes from the resting cAMP level (mean = 0.09 ± 0.03 μM). Group I metabotropic glutamate receptors (mGluR1/5) were activated with (S)-3,5-dihydroxyphenylglycine (DHPG).

**Figure 3:**
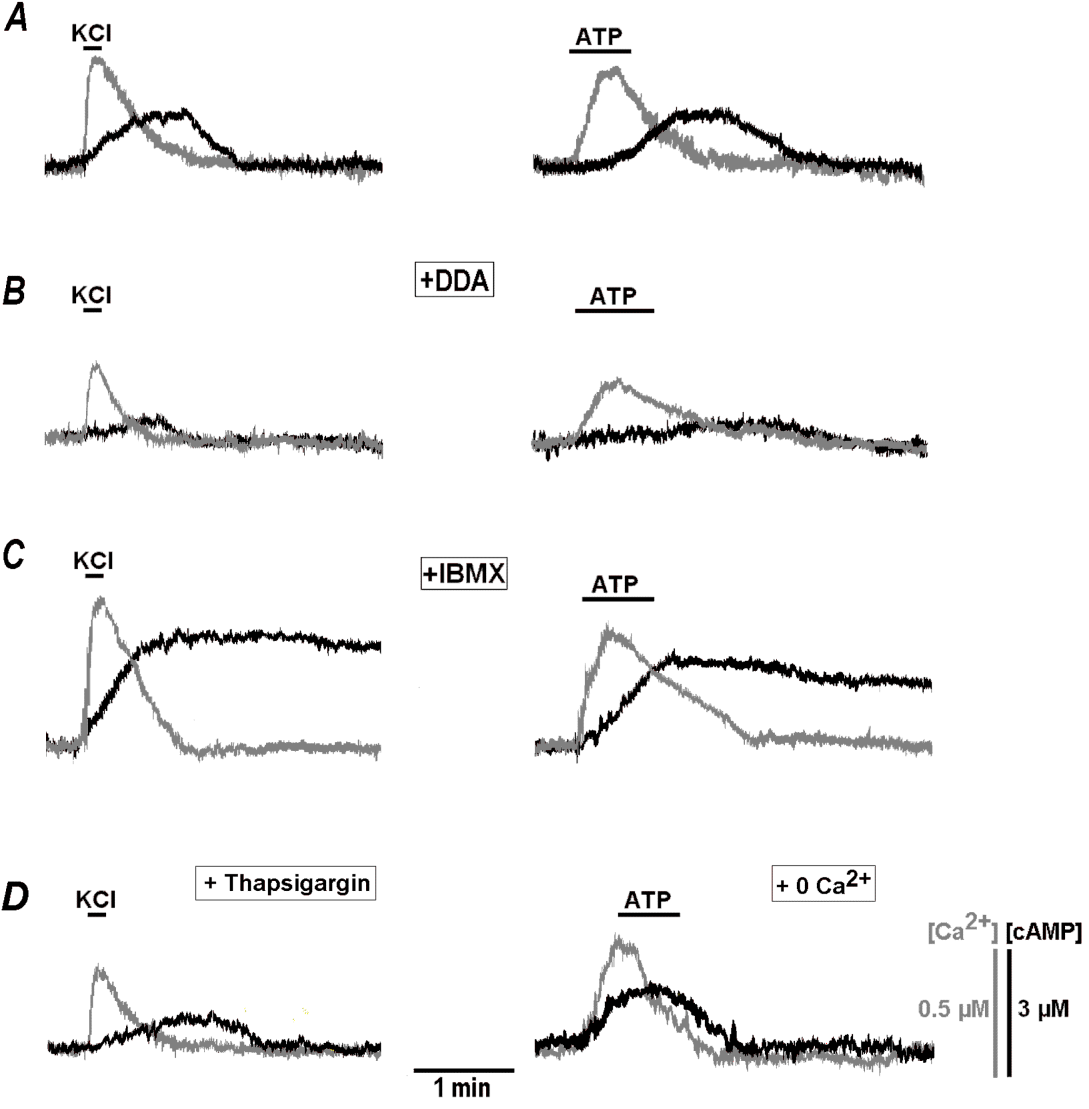
**Interrelationships between calcium and cAMP transients measured with fura-2 and Epac-1-camps**. Cytoplasmic calcium and cAMP (grey and black traces, respectively) were recorded with fura-2 and Epac-1-camps during testing challenges to 50 mM K^+ ^and 1 mM ATP as indicated. Mean peak responses are given in Table 2. The applications were made in control (A) and then in the presence of 0.1 mM DDA (B) and 50 μM IBMX (C) to inhibit the activity of adenylate cyclase and phosphodiesterase, respectively. Note the changes in the amplitude of both calcium and cAMP transients after the treatments. The two panels in (D) show the responses in [Ca^2+^]_i _and [cAMP]_i _which were recorded 10 min after preincubation with 1 μM thapsigargin to deplete calcium stores (left) and in Ca^2+^-free solution to inhibit calcium influx (right).

cAMP-signalling pathways are often activated by increases of calcium levels [1,24]. In order to examine crosstalk between calcium and cAMP, we loaded the AAV transduced neurons with fura-2 [25] and evoked calcium changes by applying brief membrane depolarisations with high-K^+ ^or by inducing calcium release from internal stores. The first paradigm is similar to the tetanic stimulation often used in the analysis of neuronal plasticity [24]. Fig. 3A illustrates how [cAMP]_i _started to increase at the peak of the calcium transient and reached its maximum during recovery of calcium levels to the resting value. Calcium release from ER induced by activation of metabotropic P_2Y _receptors with 1 mM ATP [26] also led to delayed [cAMP]_i _increases. Their amplitude and duration were similar to those induced by membrane depolarisation (Fig. 3A).

Various isoforms of AC and PDE are known to be differentially activated by calcium [21]. Ca^2+^-dependent stimulation of PDE would decrease [cAMP]_i_, therefore the effects observed indicate a dominating role of AC modulation in Ca^2+^-dependent increases. After blockade of AC activity with DDA, the amplitude of [Ca^2+^]_i _transients became smaller (Fig. 3B, Table 2), indicating that a decrease in production of cAMP can promote dephosphorylation of Ca^2+ ^channels leading to a decrease in their activity [28]. Conversely, calcium transients were significantly increased after a blockade of PDE (Fig. 3C, Table 2) that causes stimulation of PKA and subsequent phosphorylation of the channels (see also below).

**Table 2:** Mean peak [Ca^2+^]_i _and [cAMP]_i _increases due to membrane depolarisation and Ca^2+ ^release applications in the presence of drugs modulating cAMP and Ca^2+ ^homeostasis.

	Intracellular variable	Control	IBMX 50 μM	DDA 100 μM	Thapsigargin 2 μM	0 Ca^2+^
High-K^+^ 50 mM	[Ca^2+^]_i_	0.44 ± 0.07	0.72 ± 0.08	0.24 ± 0.06	0.31 ± 0.05	0.01 ± 0.01
	
	[cAMP]_i_	1.52 ± 0.09	3.22 ± 0.17	0.25 ± 0.02	0.94 ± 0.08	0.04 ± 0.01

ATP 1 mM	[Ca^2+^]_i_	0.39 ± 0.07	0.52 ± 0.08	0.24 ± 0.06	0.01 ± 0.01	0.31 ± 0.05
	
	[cAMP]_i_	1.44 ± 0.08	2.24 ± 0.07	0.36 ± 0.03	0.04 ± 0.01	1.42 ± 0.08

Mean data ± S. E. M. were obtained in 4 to 8 neurons in four different preparations. Thapsigargin and Ca^2+^-free solution were applied for 10 min before the treatments.

Removal of extracellular Ca^2+ ^or introduction of 0.1 mM Cd^2+^, which blocks all pathways for Ca^2+ ^entry into the cell, abolished depolarisation-induced [cAMP]_i _changes (Table 2). The blockade of Ca^2+ ^entry did not modify the effects of ATP (Fig. 3D). This indicates that only calcium release after activation of metabotropic P_2y _receptors is important and ionotropic P_2X _receptors are not involved in ATP-induced [cAMP]_i _increases. The effects were occluded after depletion of ER with thapsigargin (Table 2). This treatment also reduced the effects caused by membrane depolarisations by about 1/3 (Fig. 3D, Table 2) indicating contribution of Ca^2+^-induced Ca^2+ ^release (CICR [29]) to [cAMP]_i _responses.

It was recently found in presynaptic boutons of Drosophila neurons [30] that Ca^2+^-related Epac1-camps responses can be mediated by changes in [cGMP]_i_. This apparently contradicts the fact that the affinity of the sensor to cGMP (K_d _= 11 μM) is by about one order of magnitude lower than that for cAMP [12]. However, the presynaptic calcium transients may have considerably higher amplitude and cause AC inactivation (see Discussion) which would make [cGMP]_i _changes dominant. In order to examine whether cGMP changes can underlie, in part, the responses of Epac1-camps, we applied guanylyl cyclase inhibitor 1H-[1,2,4]-oxadiazolo- [4,3-a]-quinoxalin-1-one (ODQ, 100 μM), the NOS inhibitor N-monomethyl-L-arginine (L-NMMA), and the NO donor S-nitroso-N-acetylpenicillamine (SNAP, 300 μM). These drugs were applied at concentrations at which they showed distinct effects on the respiratory motor output *in vivo *[31], but they did not modify the signals of Epac1-camps (*n *= 3 for each treatment).

**Figure 4:**
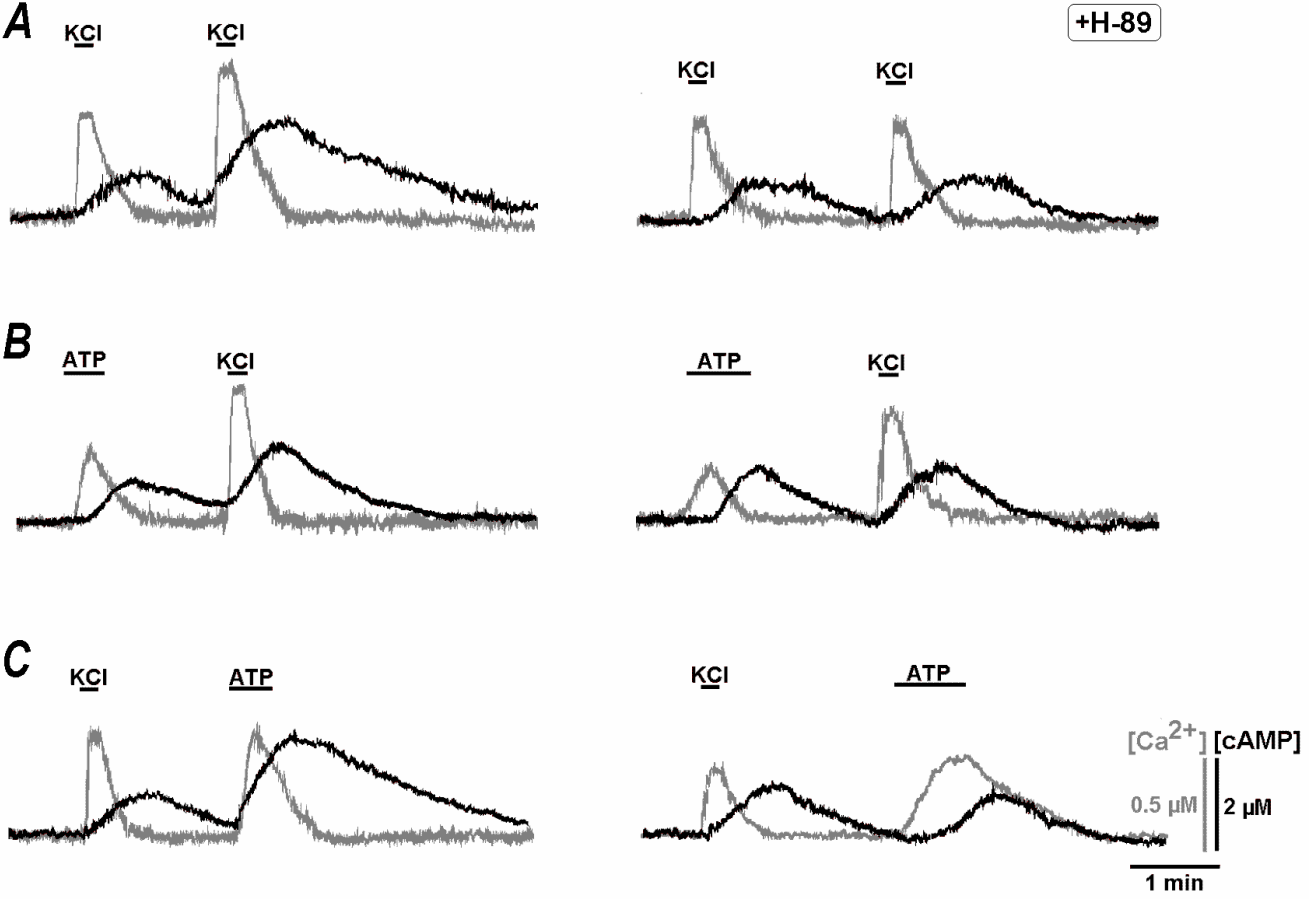
**Non-linear interactions between Ca^2+ ^influx and Ca^2+ ^release during [cAMP] increases**. Testing applications of 50 mM K^+ ^and 100 μM ATP were made as indicated by horizontal bars. Changes in [Ca^2+^]_i _and [cAMP]_i _are shown by grey and black traces, respectively. The experiments were performed in control (*left*) and repeated 20 min after pretreatment with protein kinase A inhibitor H-89 (10 μM, *right*). A – Two depolarisation with high K^+^. B – Ca^2+ ^release from ER with following depolarisation. C – Depolarisation and Ca^2+ ^release. Note that potentiation of [cAMP] increases during the second treatments was abolished by H-89.

We observed a mutual interference between Ca^2+ ^and cAMP signalling pathways. Application of paired stimuli produced bigger [cAMP]_i_responses during the second stimulation which increased calcium (Fig. 4A). Such enhancement of the response can be mediated by Ca^2+^-driven stimulation of PKA increasing the activity of voltage-sensitive Ca^2+^channels [4,26]. In order to test this assumption, we applied PKA inhibitor H-89 (*N*- [2-(*p*-bromocinnamylamino)ethyl]-5-isoquinoline sulfonamide hydrochloride, 10 μM for 10 min). After pretreatment with H-89, the potentiation of secondary [Ca^2+^]_i _and [cAMP]_i_increases was abolished (Fig. 4A). The enhancement of [cAMP]_i _responses was also observed after calcium release from ER and corresponding effects were inhibited by H-89 (Fig. 4B, C).

## Discussion

Many vitally important processes which determine the life and fate of neurons are controlled by cAMP and Ca^2+^, which often act in parallel [1]. In non-excitable cells the fluctuations of [Ca^2+^]_i _and [cAMP]_i _are often interrelated [32] and linked via adenylate cyclase [21] or phosphodiesterase [21]. AC has eight different isoforms which can be stimulated and inhibited by Ca^2+^. The stimulation is characteristic for AC1 and AC8 which are abundant in the brain and activated by calmodulin. All AC isoforms are inhibited by supramicromolar concentrations of Ca^2+ ^(> 10 μM) binding to a low-affinity site in the catalytic AC domain [21]. We recorded calcium transients of much smaller amplitude, therefore a Ca^2+^-mediated inhibition of AC should not play any significant role in the effects reported here. It may however become significant in domains which are located close to the exit of Ca^2+ ^from the channels producing calcium gradients which can reach values as high as 100 μM [33] and locally limit the production of cAMP.

The levels of Ca^2+ ^and cAMP are often changed by various neurotransmitters and [cAMP]_i _changes may be further differentially decoded downstream through, for example, PKA, Epac, cAMP-binding protein (CREB) and Rap to initiate events such as gene expression and cell differentiation. PKA-mediated phosphorylation of channels is important for normal functioning of the channels that mediate entry of Ca^2+ ^into the cytoplasm and its release from internal stores. These phosphorylation/dephosphorylation reactions thus close a regulatory Ca^2+^/cAMP feedback.

In comparison to Ca^2+^, we currently know much less about the changes in intracellular cAMP which can occur in relation to neuronal activity. Increasing applications of cAMP sensors developed in the last decade [8-12] to different neuronal preparations will definitely bring new knowledge in this area. Although until now such studies have been made only in some cell lines [34] and isolated neurons in primary culture [35], they have already revealed the complexity of cAMP dynamics. The neurons in living tissue present the next level of sophistication, as their properties can be modified by interactions with neighbouring neurons and surrounding glial cells. Intrinsic electrical activity of neurons add another important factor, because ensuing calcium fluxes of different amplitude and time-courses and spontaneous calcium release from internal stores should substantially modulate spatial [cAMP]_i _patterns on different time scales. How they are involved in different vital cellular functions such as neuronal plasticity, differentiation and development remains to be established.

Currently available cAMP sensors are bulky proteins and the first obstacle in cAMP imaging concerns the delivery of the probe into the cytoplasm. A straightforward way is a single cell injection [8] with well known difficulties. Transgenic animals would be an ideal solution and this approach has been successfully used to study cAMP signalling in the heart [23], pancreatic islets [36] and the neurons of fruit-flies [37]. Non-cytotoxic viral gene transfer presents an alternative not limited in its application by the animal or tissue [15,38]. It can be applied to any cell population given that the sensor is targeted to the specific cell type. We used a strictly neuron-specific promoter [15] to deliver the cAMP sensor [12] into neurons in slices. As a proof of concept, we showed that Epac1-camps is expressed in many neurons and reports [cAMP]_i _levels and their fluctuations in μM range after various physiological stimuli. We observed that membrane depolarisation and calcium release from internal stores produced [cAMP]_i _increases that led to further enhancement of calcium entry or calcium release through PKA-dependent phosphorylation. The effects were observed after relatively long-lasting calcium increases similar to those evoked by the tetanic stimulation commonly used to induce a long-term potentiation [24]. The concerted actions of the two second messengers can convey specific messages between neurons but Ca^2+ ^and cAMP should have different spatial and temporal ranges of actions. For example, the production and consumption rates of cAMP as well as its diffusion coefficient (Nikolaev et al. 2004) are more consistent with slower and spatially extended changes, whereas the actions of Ca^2+ ^must be faster and more localised. How the effects of these two second messengers are orchestrated, and how they influence the neuronal activity, represents a challenging task for future studies. We believe that neuronal specificity, optical stability and sensitivity of Epac1-camps-based sensor provide a solid platform for such examinations.

## Conclusion

We here report the application of Epac1-camps to measure cAMP concentrations in mammalian neurons embedded in their three dimensional context in living tissue. The sensitivity and specificity of neuron-targeted Epac1-camps are characterized and the probe was applied to examine crosstalk between calcium and cAMP signalling in brainstem neurons.

## Methods

All animals were housed, cared for and euthanized in accordance with the recommendations of the European Commission (No. L358, ISSN 0378-6978), and protocols approved by the Committee for Animal Research, Göttingen University. Organotypic culture slices were obtained as described previously [16]. Briefly, we prepared 250 μm-thick brainstem slices following a conventional procedure to obtain so called 'rhythmic slices' [17,20,30,37]. All slices contained typical anatomy markers as documented in the atlas of the brainstem [19].

After preparation slices were placed on support membranes (Millicell-CM Inserts, PICMORG50; Millipore). 1 ml medium added to let the surface of the slice be continuously exposed to the incubator gas mixture and the medium (50% MEM with Earle's salts, 25 mM Hepes, 6.5 mg/ml glucose, 25% horse serum, 25% Hanks solution buffered with 5 mM Tris and 4 mM NaHCO_3_, pH 7.3) changed every second day. During the experiments, each slice was fixed on a coverslip mounted in the recording chamber and was continuously superfused at 34°C with artificial cerebro-spinal fluid (ACSF). Under experimental conditions the bath solution was fully exchanged within 1 s for different periods of time ranging from 10 s (applications of 50 mM K^+^, 100 μM ATP) to 2 min (forskolin, IBMX, DDA, rolipram etc.). High-K^+ ^solution was prepared by exchanging Na^+^in ACSF; all other testing solutions were produced by adding aliquots of corresponding stock solutions directly to ACSF at approx. 1000-fold dilution.

Neurons in slices were transduced with AAV-Epac1-camps which expressed the sensor under control of the strictly neuron-specific synapsin 1 gene promoter [15]. 1 μl of AAV solution (≈ 1 × 10^9 ^viral genomes) was applied directly onto the slice surface. Epac1-camps expression reached a steady state two days after viral application and did not change afterwards. Each test in the study was repeated with at least four different preparations, 4 to 8 neurons in each image field were examined.

The optical recording system was based on a Zeiss Axioscope and the slices were viewed under a 10× (Achroplan, N. A. 0.10) or 40× objective (Achroplan, N. A. 0.8). Epac1-camps was excited at 430 nm by the light generated by LED (20 mW, Roithner Lasertechnik). CFP emission at 470 nm and FRET between CFP and YFP (emitted at 535 nm) were separated with Optosplit (BFI Optilas, Puchheim) using dichroic mirror at 495 nm and 470 ± 12 and 535 ± 15 nm filters, respectively. Bleaching of Epac1-camps was negligible, thus allowing us to record fluorescence for more than 60 min without substantial loss of the signal. Images were captured by a cooled CCD camera (ANDOR, Offenbach) and collected with ANDOR software (500 × 500 pixels at 12 bit resolution). Time-dependent changes were obtained offline using MetaMorph software (Princeton Instruments, USA). The data were collected in the regions of interest which encircled the soma of neurons.

**Figure 5:**
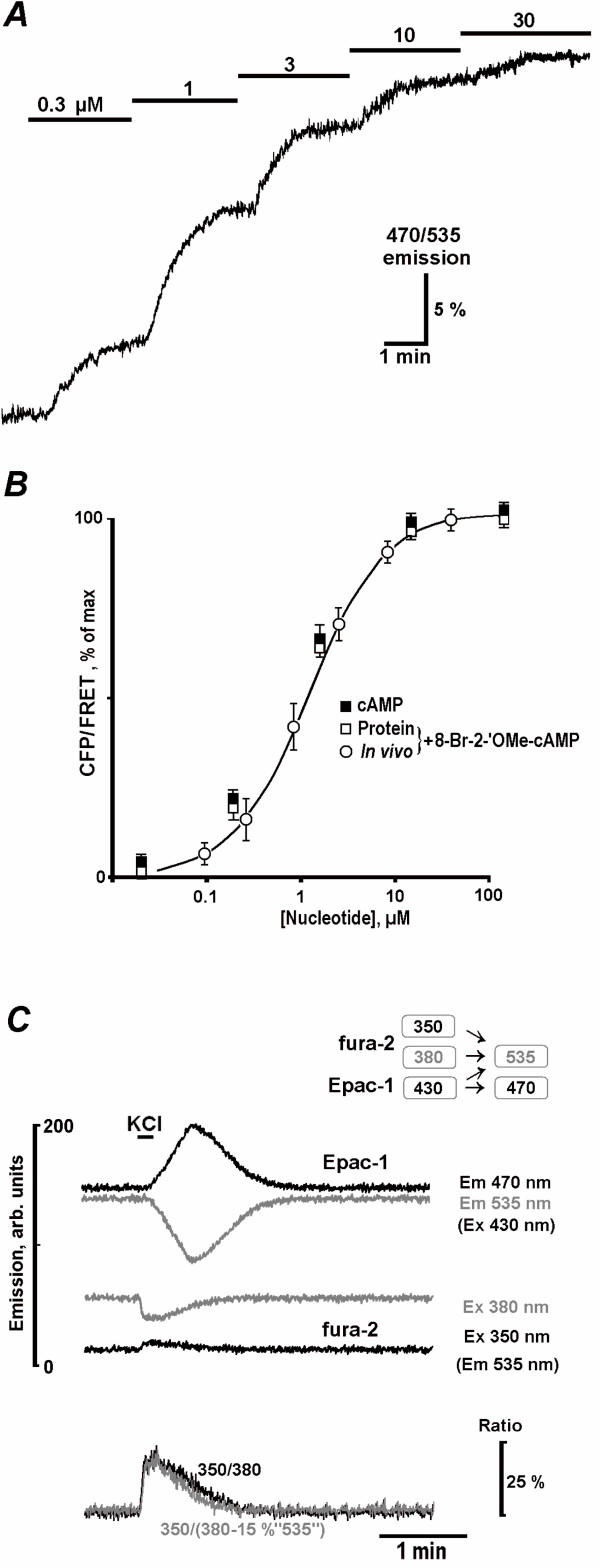
**Calibration of cAMP levels in neurons**. A****– Increases in 470/535 nm emission ratio during sequential additions of specific membrane-permeable Epac agonist 8-Bromo-2'-OMe-cAMP. B – Calibration of Epac-1-camps. Mean ratios ± S. E. M were obtained for 8-Bromo-2'-OMe-cAMP *in vivo *and *in vitro*, and for cAMP *in vitro*. The data are plotted against nucleotide concentration and the smooth curve was drawn according to the Michaelis – Menten-like equation with *K*_*d *_= 1.5 μM. C – Typical Ca^2+^-dependent changes in [cAMP] after membrane depolarisation. Shown are the raw traces of Epac-1-camps emission at 470 nm (CFP) and 535 nm (FRET) excited at 430 nm and of fura-2 emission at 535 nm excited at 350 and 380 nm as indicated. The lower two traces demonstrate changes in the fura-2 ratio at excitation wavelengths 350 and 380 nm and those obtained after subtraction of 15% of Epac1-camps signal (Ex 430 nm, Em 535 nm) from fura-2 signal (Ex 380, Em 535 nm).

[cAMP] values were obtained from the ratio of Epac1-camps fluorescence (R) excited at 430 nm and emitted at 535 nm (FRET) and 470 nm (CFP). Fig. 5A presents calibration of cAMP levels *in vivo *using membrane-permeable 8-Bromo-2'-O-methyl-cAMP (BrOMecAMP), a specific activator of Epac [39]. Measured ratio changes (*R*) were well fitted with the Michaelis-Menten-like equation

(1)

where *C *is the concentration of nucleotide and *K*_*d *_is the dissociation constant for Epac1-camps (Fig. 5B). In estimating *R*_*max *_we accounted for the basal [cAMP] level by suppressing AC activity with DDA (see Results). Absolute cAMP levels were obtained as

(2)

The basal [cAMP]_i _was 0.09 ± 0.03 μM (*n *= 16), corresponding to the lower branch of the dose-response curve in Fig. 5B, and all [cAMP]_i _observed changes were well below 5 μM (Tables 1 and 2), indicating that the probe has an optimal dynamic range for imaging cAMP in neurons. From Eqs. (1) and (2) follows that [cAMP] is approximately proportional to the measured ratio for [cAMP] levels when they are below *K*_*d *_of the sensor (1.5 μM). Because this condition is not always fulfilled, presentation of [cAMP] changes in the ratio form may show erroneous kinetics.

Epac1-camps also was calibrated *in vitro *using previously described procedures [12]. Briefly, HEK293 cells were transfected with Epac1-camps. 24 h after transfection, cells were washed three times and resuspended in 5 mM Tris, 2 mM EDTA, pH 7.4 buffer. After disruption with an Ultraturrax device for 40 s on ice and 20 min centrifugation at 80000 rpm, fluorescence emission spectra of the supernatant (excitation at 436 nm, emission range 460 – 550 nm) were measured with a fluorescence spectrometer LS50B (Perkin Elmer Life Sciences) before and after addition of cAMP and BrOMecAMP (Biolog Life Science Institute, Bremen, Germany) at various concentrations. The concentration of Epac1-camps protein was 50 nM. FRET/CFP ratios were calculated by dividing peak emission intensities at 524 nm (FRET) and 477 nm (CFP) and analyzed by Origin 6.1 (Origin Lab Corporation, Northampton, MA). The use of emission wavelengths with wider slits such as set up in an Optosplit gave similar ratios. The curves for BrOMecAMP *in vivo *and *in vitro *were identical and were close to the dose-response curve obtained for the sensor in slices (Fig. 5B). *K*_*d *_values *in vivo *and *in vitro *were 1.6 and 1.5 μM for BrOMecAMP, respectively, and 1.2 μM for cAMP.

For imaging of intracellular calcium, 3 μM fura-2/AM was added to ACSF as an aliquot of DMSO-based stock solution and the mixture was sonicated. Slices were incubated with the dye for 20 min at 37°C followed by a 30 min-long wash-out to allow deesterification of ester precursor. Fura-2 was excited at 350 and 380 nm, and the emission was collected at 535 nm. [Ca^2+^]_i _values were obtained as described previously [40].

During simultaneous calcium and cAMP imaging we made concerns on possible contamination of the recorded fura-2 signal by CFP fluorescence from Epac1-camps which can be also excited by UV [25]. Fig. 5C shows the two pairs of Epac1-camps (470 and 535 nm emission at 430 nm excitation) and fura-2 signals (535 nm emission at 350 and 380 nm excitation), which were recorded during high-K^+ ^stimulation. Imaging of cells which were not loaded with fura-2 showed that excitation of CFP at 380 nm produced the fluorescence at 535 nm which is only 10 ± 2% (*n *= 24) of that recorded at 430 nm excitation. This estimate agrees well with the excitation spectrum of Epac1-camps *in vitro *[12]. The two lowermost traces in Fig. 5C show the effect of possible bleeding of Epac1-camps fluorescence into the fura-2 channel. Subtraction of 15% Epac1-camps signal (Ex 430 nm, Em 535 nm) taken as an upper limit for bleeding to fura-2 channel (Ex 380 nm, Em 535 nm) did not change the traces significantly.

## Abbreviations

AAV: Adeno-associated virus; AC: adenylate cyclase; ACSF: artificial cerebro-spinal fluid; BrOMecAMP: 8-Bromo-2'-OMe-cAMP; [Ca^2+^]_i_: cytoplasmic free Ca^2+^; cyan (yellow) fluorescent protein: CFP (YFP); DDA: 2'5'-dideoxyadenosine: Epac: cAMP-dependent exchange factor; ER: endoplasmatic reticulum; FRET: fluorescence resonance energy transfer; IBMX: isobutylmethylxanthine; H-89: *N*- [2-(*p*-bromocinnamylamino)ethyl]-5-isoquinoline sulfonamide hydrochloride; PDE: phosphodiesterase; PKA: protein kinase A; preBötC: pre-Bötzinger complex.

## Authors' contributions

ES performed the imaging and carried out the statistical analysis, NH designed and produced organotypic slice preparation, VON designed cAMP sensor, performed its calibration *in vitro *and helped to draft the manuscript, MJL participated in the design of the sensor, GT and SK generated the recombinant AAV-Epac-1-camps virus, SK participated in the design of the study and writing the manuscript, SLM conceived, designed and coordinated the study, performed the imaging, carried out the analysis of the experimental data and wrote the manuscript. All authors have read and approved the final manuscript.
